# Lipid metabolism dysregulation in diabetic retinopathy

**DOI:** 10.1194/jlr.TR120000981

**Published:** 2021-01-06

**Authors:** Julia V. Busik

**Affiliations:** Department of Physiology, Michigan State University, East Lansing, MI, USA

**Keywords:** fibrate, statin, triglyceride, cholesterol, ceramide, mitochondria, tight junctions, fatty acid, ASM, acid sphingomyelinase, BRB, blood-retinal barrier, DR, diabetic retinopathy, RPE, retinal pigment epithelial, SC, short chain, TJ, tight junction, VLC, very long chain

## Abstract

Lipid metabolic abnormalities have emerged as potential risk factors for the development and progression of diabetic complications, including diabetic retinopathy (DR). This review article provides an overview of the results of clinical trials evaluating the potential benefits of lipid-lowering drugs, such as fibrates, omega-3 fatty acids, and statins, for the prevention and treatment of DR. Although several clinical trials demonstrated that treatment with fibrates leads to improvement of DR, there is a dissociation between the protective effects of fibrates in the retina, and the intended blood lipid classes, including plasma triglycerides, total cholesterol, or HDL:LDL cholesterol ratio. Guided by these findings, plasma lipid and lipoprotein-independent mechanisms are addressed based on clinical, cell culture, and animal model studies. Potential retinal-specific effects of fatty acid oxidation products, cholesterol, and ceramide, as well as lipid-independent effects of PPAR alpha activation, are summarized based on the current literature. Overall, this review highlights promising potential of lipid-based treatment strategies further enhanced by the new knowledge of intraretinal lipids and lipoproteins in DR.

Hyperglycemia is well accepted as a major risk factor for the development of microvascular diabetic complication, including diabetic retinopathy (DR), and clinical trials unequivocally demonstrated a strong link between the improvement of glycemic control and reduction of the onset and progression of diabetic retinopathy in both type 1 (T1D) (1) and type 2 (T2D) (2) diabetes. In addition to hyperglycemia, lipids and lipoproteins have been recently proposed to contribute to the pathogenesis of DR; however, the associations of individual lipid classes with DR are variable between the studies and weak overall. This is not surprising; owing to the complexity, tissue specificity, and cross-connected nature of the lipid metabolism, the role of individual lipid classes in disease is hard to evaluate. Intervention studies with fibrates (3, 4, 5, 6) and omega-3 fatty acids (7) have demonstrated variable degrees of protection against the development and progression of DR. The improvement of DR observed with fibrates was independent of the intended blood lipid classes, raising the questions about tissue-specific, lipid-independent, or different lipid class mechanisms (Fig. 1). The following sections address the potential mechanisms of lipid-mediated DR pathogenesis beyond traditional blood triglyceride and HDL/LDL cholesterol levels.

**Fig. 1:**
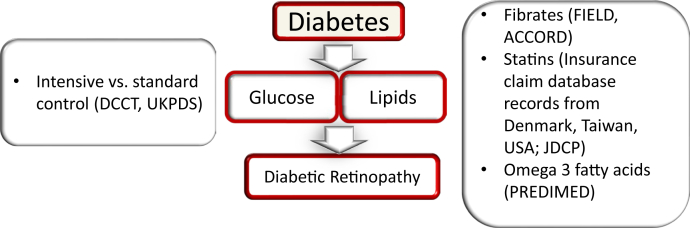
Clinical trials and large database studies that shaped our understanding of the risk factors for the development and progression of diabetic retinopathy. The role of hyperglycemia was first established in the DCCT and UKPDS (UK Prospective Diabetes Study) clinical trials (left). The role of lipid metabolism dysregulation is suggested based on the FIELD, ACCORD, and PREDIMED clinical trials and the Denmark, Taiwan, US, and Japan database studies (right).

## Role of Lipids in DR

Although hyperglycemia, duration of diabetes, high blood pressure, and microalbuminuria are known to confer higher risk for the development and progression of DR, it has become clear that there are additional, yet unidentified risk factors for the disease. Plasma lipids and lipoproteins have been proposed as potential risk factors for DR and especially for hard exudates for several decades. Indeed, lipid-lowering dietary (8) and fibrate (9) therapy studies demonstrated regression of retinal hard exudates, and a diet high in polyunsaturated fatty acids suggested protection against retinopathy (10). Several clinical trials and epidemiological studies have demonstrated a positive association between plasma LDL and DR (11, 12, 13, 14, 15, 16, 17, 18, 19, 20, 21, 22, 23, 24, 25, 26, 27). Early cross-sectional studies demonstrated positive associations between the severity of retinopathy and total- and LDL-cholesterol levels and LDL:HDL cholesterol ratio (12). The first large-scale clinical trial data came from the Diabetes Control and Complications Trial (DCCT)/Epidemiology of Diabetes Interventions and Complications (EDIC) cohort, which revealed strong associations between the severity of retinopathy in T1D and the size of the particles of three major classes of serum lipoproteins—very low density, low density, and high density lipoprotein (VLDL, LDL, and HDL)—as well as LDL concentration (26). The Early Treatment Diabetic Retinopathy Study (ETDRS) also demonstrated that higher levels of serum lipids are associated with an increased risk of development of hard exudates in the macula and visual loss (24, 28).

## Benefits of lipid-lowering drugs

### Fibrates

Unlike the large well-designed, long-term clinical trials on the effect of intensive glycemic control and HBA1c levels that had diabetic retinopathy as a primary outcome (1, 2), most of the trials on lipid-lowering drugs were designed as cardiovascular trials with microvascular complications, including diabetic retinopathy, as secondary or tertiary outcomes. As DR was not a primary target, several key outcome measures for DR were missing and the DR outcomes were not standardized between the trials.

Perhaps the most well-known benefit of the lipid-lowering drugs in DR came from two cardiovascular trials of T2D, Fenofibrate Intervention and Event Lowering in Diabetes (FIELD) (6) and Action to Control Cardiovascular Risk in Diabetes (ACCORD) (3, 5, 29) studies. In FIELD, patients were treated with peroxisome proliferator-activated receptor (PPAR)α agonist (fibrate), fenofibrate, at 200 mg/day, or matching placebo. Statin treatment was an exclusion criterion at the recruitment stage of FIELD; however, a significant number of patients started statin treatment during the study. Information on laser treatment for diabetic retinopathy was a prespecified tertiary end point of the main FIELD study and was collected at each visit; however, more detailed standardized retinal photography and grading with the ETDRS scale was performed only in a substudy of 1,012 patients. The FIELD study demonstrated a reduced frequency of laser photocoagulation by 30% and 31% for proliferative DR and diabetic macular edema, respectively (6).

The substudy with more detailed outcomes further showed that, in the group with preexisting retinopathy, significantly fewer patients on fenofibrate had a two-step progression than did those on placebo; however, this effect was not observed in the patients without preexisting retinopathy. Previous, much smaller-scale studies of fibrates also found beneficial effects on retinal (30, 31, 32, 33) and macular hard exudates (9, 34). In FIELD, the effects of fibrates on DR pathogenesis were unrelated to its effects on plasma triglycerides, HDL and LDL cholesterol, suggesting that the DR effects might be attributable to a specific, intraretinal action.

The ACCORD study evaluated the effects of intensive versus standard control of blood glucose levels, serum lipid levels, and blood pressure on cardiovascular events in participants with T2D who had either established cardiovascular disease or known cardiovascular risk factors. A subgroup of 2,856 participants with preexisting simvastatin treatment was randomized to fenofibrate versus placebo (ACCORD-Eye). This group had two comprehensive standardized eye examinations and fundus photography performed at baseline and year 4 of follow-up, and the effects of the treatments on the progression of diabetic retinopathy were determined by three or more steps on the ETDRS scale or the development of diabetic retinopathy necessitating laser photocoagulation or vitrectomy. The study confirmed the importance of tight glycemic control and demonstrated a significantly reduced rate of DR progression after 4 years in those receiving fenofibrate and simvastatin (6.5%) versus placebo and simvastatin (10.2%), (adjusted odds ratio, 0.60; 95% CI, 0.42–0.87; *P* = 0.006) (3). As with the FIELD study, there was a dissociation between the protective effects of fenofibrate in the retina and plasma triglycerides, total cholesterol, or HDL:LDL cholesterol ratio (3, 5), again suggesting a specific, intraretinal action.

### Omega-3 fatty acids

In addition to using fibrates, several cardiovascular trials used different formulations of omega-3 fatty acid as the route for PPAR activation and control of triglyceride levels in the patients with diabetes. In the ORIGIN (Outcome Reduction with Initial Glargine Intervention) study, patients were supplemented with 900 mg of ethyl esters of omega-3 fatty acids (90% or more) or placebo daily. Supplementation with omega-3 fatty acids did not reduce the rate of cardiovascular events in patients at high risk for cardiovascular events.

In the ASCEND trial (A Study of Cardiovascular Events in Diabetes), patients were supplemented with 460 mg of eicosapentaenoic acid and 380 mg of docosahexaenoic acid or a matching olive oil placebo capsule once daily for an average of 7.3 years duration. There was no significant reduction in primary composite end points; however, cardiovascular disease death was significantly reduced by 19%.

In REDUCE-IT (Reduction of Cardiovascular Events with Icosapent Ethyl–Intervention Trial), patients were supplemented with 2 g eicosapentaenoic acid (icosapent ethyl) twice daily for an average of 4.9 years. The supplementation resulted in a 25% decrease in the primary end point of major cardiovascular events within patients with elevated triglycerides (135–499 mg/dL) who also were taking a statin drug.

Microvascular outcomes were included as secondary outcomes in these cardiovascular trials. The ASCEND reported no difference in diabetic retinopathy outcomes based on patient self-reporting. No other microvascular outcomes were observed.

The PREDIMED (Prevención con Dieta Mediterránea) study demonstrated that an increased dietary intake of long-chain omega-3 polyunsaturated fatty acids (at least 500 mg/day) was associated with a nearly 50% relative risk reduction for vision-threatening diabetic retinopathy among older individuals with T2D (7). Variable outcomes between the trials are likely due to the difference in study designs, the doses of interventions, and the composition of treatment. ORIGIN, ASCEND, and REDUCE-IT used capsule supplementation, whereas PREDIMED used dietary intake with the diets enriched in fish and nuts. As a Mediterranean study, PREDIMED benefited from a population whose dietary recommendations were easier to achieve, as fish and nuts are contained in many staple dishes in the area.

### Statins

Although there is a consensus on the benefits of fibrates in DR prevention, the role of statins is more controversial. Simvastatin alone did not result in further protection in the ACCORD study (3), and statins have proven unsuccessful in preventing DR in a small trial (35); however, large database studies from Denmark, Taiwan, and recently the United States showed a potential role of statins. The Danish Patient Registry and information on drug use from the Danish Registry of Medicinal Product Statistics showed that patients who used statins before the diagnosis of diabetes had a lower cumulative incidence of diabetic complications, including diabetic retinopathy, compared with nonstatin users, suggesting a potential protective effect (36). The Taiwan National Health Insurance Research Database study compared risk between patients with and without statins use. Statin therapy was associated with a decreased risk of diabetic retinopathy and need for treatments for vision-threatening diabetic retinopathy (37). To evaluate the impact of lipid-lowering medications on diabetic retinopathy and diabetic complications requiring intervention in the US population, administrative insurance claims were drawn from the Truven MarketScan Commercial Claims and Encounters databases. The study found consistent evidence that patients taking lipid-lowering medications were less likely to develop non-proliferative DR, proliferative DR, or diabetic macular edema and modest evidence that these patients are less likely to receive intravitreal injections of anti-vascular endothelial growth factor medication, laser treatments, or vitrectomy (38).

The Japan Diabetes Complication and its Prevention prospective (JDCP) study, a nation-wide study capturing real-world practice for diabetes in Japan, recruited patients with T1D and T2D aged between 40 and 75 years. Statin and fibrate use was associated with lower odds of having non-proliferative DR; this association was confirmed in the model adjusting for the propensity score for taking fibrate or statin (39).

The dissociation between the retinal effects of fibrates, and potentially statins, and those on blood lipid levels, led to several hypotheses that are being addressed. First, intraretinal lipid transport rather than serum lipid concentrations was proposed to contribute to the pathogenesis of diabetic retinopathy (40). The blood-retinal barrier breakdown that occurs early in the pathogenesis of DR allows for the increase in the nonspecific LDL entry and increased retinal levels. This is further exacerbated by decreased retinal cholesterol efflux in diabetes (discussed below). In the diabetic environment, this could lead to increased glycation and oxidation of LDL. Oxidized glycated LDL has prominent proinflammatory and proatherogenic effects (41, 42): in retinal cell studies, it induces Muller cell activation (43) and is cytotoxic to retinal capillary pericytes (44) and to retinal pigment epithelial (RPE) cells (45). In addition, oxidized LDL immunocomplexes were implicated to play a role in diabetic retinopathy (46). The product of free radical oxidation of cholesterol, 7-ketocholesterol, is elevated in diabetes and has potent proapoptotic and proinflammatory properties (47, 48).

## Potential retinal-specific mechanisms: what we can learn from animal and cell culture data

### Role of fatty acids and oxidized fatty acid products in DR pathogenesis

**Fig. 2:**
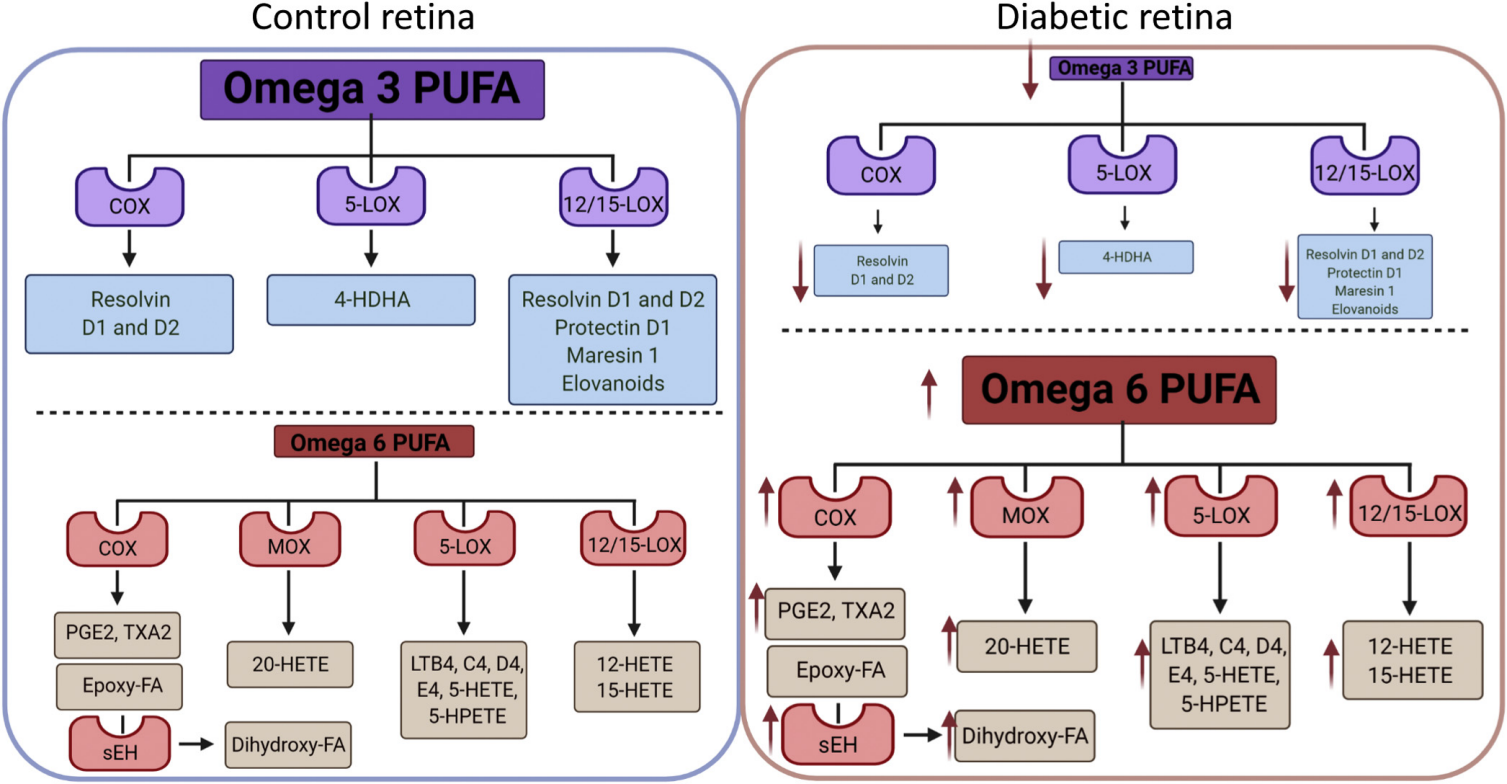
Fatty acids and oxidized fatty acid products in control and diabetic retina. Control retina is enriched in omega-3 PUFA with lower levels of omega-6 PUFA (left panel). Diabetes shifts the balance between PUFA toward lower omega-3 PUFA and higher omega-6 PUFA (right panel). This leads to a change in composition of oxidized fatty acid products with a shift from omega-3 PUFA-derived proresolution products, resolvins and protectins, to omega-6 PUFA-derived proinflammatory monooxygenase (MOX), lipoxygenase (LOX), and COX oxidized lipid mediators. Hydrolysis of COX PUFA metabolites by soluble epoxide hydrolase gives rise to dihydro-FAs that further contribute to the pathogenesis of DR.

Several animal and cell culture studies demonstrated that the highest retinal omega-3 polyunsaturated fatty acid (PUFA)-docosahexaenoic acid (DHA) has pronounced antiinflammatory, antiapoptotic effects in the retina and retinal cells (49, 50, 51, 52, 53, 54, 55, 56). A diabetes-induced decrease in DHA with concomitant increase in proinflammatory omega-6 PUFA (49, 50, 51, 53, 57) was shown to contribute to the development of DR through several mechanisms, including effects of omega-3 PUFA on plasma membrane and lipid rafts (51, 54), as well as a change in the composition of oxidized fatty acid products with a shift from omega-3-PUFA-derived resolvins and protectins (49, 58, 59) to omega-6 PUFA-derived monooxygenase, lipoxygenase, and cyclooxygenase (COX) oxidized lipid mediators (56, 57, 60, 61, 62, 63, 64, 65, 66). Hydrolysis of COX PUFA metabolites by soluble epoxide hydrolase was recently shown to be affected in DR and proposed as a potential therapeutic target (60, 61, 62, 63, 65, 66, 67). In addition to having direct effects, DHA is also elongated to very long-chain (VLC)-PUFAs by the action of Elovl1 and 4 (53, 68, 69). The oxidized products of VLC-PUFA, elovanoids, were recently shown to have retinal-protective effects (70, 71, 72, 73). The levels and the role of elovanoids in DR are not known. The decrease in elovanoids in the diabetic retina could be predicted based on a decrease in Elovl1 and 4 and thus VLC-PUFAs, the substrates from which elovanoids are formed (Fig. 2).

### Diabetes-induced dysregulation of retinal-specific cholesterol metabolism and DR

**Fig. 3:**
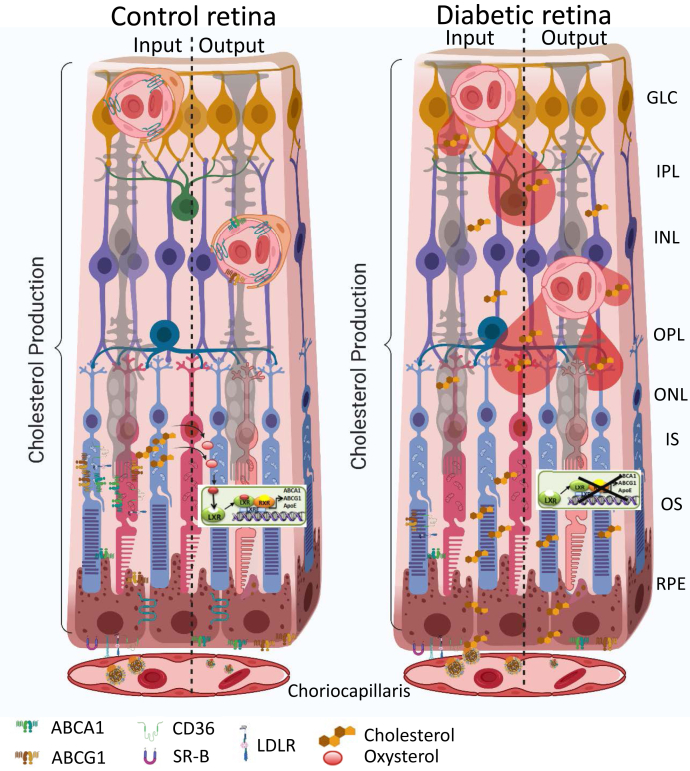
Cholesterol metabolism in control and diabetic retina. The input is controlled by the local production by most retinal cells, and uptake from circulation by RPE cells through CD36, SR-B, and LDLR. The output is through oxidation to oxysterols, LXR activation, and production of ABCA1, ABCG1, and ApoE that control cholesterol efflux through RPE and endothelial cells (left). In diabetes, owing to the blood-retinal barrier breakdown there is an increase in nonspecific cholesterol entry, and reduction of LXR activity and cholesterol efflux, leading to an increased cholesterol accumulation in the retina (right). GLC, ganglion cell layer; INL, inner nuclear layer; IPL, inner plexiform layer; IS, photoreceptor inner segment; ONL, outer nuclear layer; OPL, outer plexiform layer; OS, photoreceptor outer segment; RPE, retinal pigment epithelium.

The retina maintains its cholesterol homeostasis by tight control and balance of the pathways responsible for cholesterol input versus output (Fig. 3) (74). A more detailed overview of cholesterol homeostasis in the vertebrate retina is provided by Rao and Fliesler in this issue (75). Unique metabolic demands and the highly specialized structure and function of the retina dictate complex regulatory pathways to support retinal metabolism while preserving autonomy behind the two blood-retinal barriers (BRBs) that separate it from the systemic circulation (76, 77, 78). The retinal vascular endothelial cells connected by tight junctions forming the inner BRB, which, when intact, is impermeable to cholesterol. Breakdown of inner BRB in diabetic retina, however, could lead to nonspecific entry of lipoprotein particles into the retina, increasing retinal cholesterol levels. The outer BRB, formed by the RPE cells, allows for cholesterol transport into the retina. Cholesterol in the retina is derived either from local biosynthesis (74, 76, 77, 79, 80, 81) or from uptake of lipoprotein particles from the choroidal circulation through the outer BRB. After uptake by the RPE, cholesterol is exported by ABCA1- and ABCG1-transporters either back to the choroidal circulation by reverse cholesterol transport or to the neural retina (81, 82, 83, 84, 85, 86). In addition to exporting cholesterol, both RPE and neural retina metabolize cholesterol to more soluble oxysterols by cytochrome P450s (CYPs), 27A1, and 46A1 (81, 82, 83, 84, 85, 86, 87).

These oxysterols are activating ligands for liver X receptors (LXRs). LXR activation plays a dual role in the retina: it activates reverse cholesterol transport genes and suppresses NF-κB-mediated inflammatory gene expression. In addition to its activation by oxysterols, LXR activity is controlled by acetylation status. LXR deacetylation is mediated by a nutrient-sensing deacetylase, SIRT1, increasing LXR activity. Diabetes-induced disruption of the SIRT1-LXR axis and reduced oxysterol production due to loss of cytochromes P450, 27A1, and 46A1 in the retina result in diminished cholesterol removal leading to inadequate vascular repair, macrophage/microglia activation, and widespread retinal pathology (88, 89, 90). Activation of LXR restores reverse cholesterol transport, prevents inflammation, and prevents the formation of diabetes-induced acellular capillaries (88). Moreover, SIRT1 stimulation by fasting or by pharmacological activation using SRT1720 leads to LXR deacetylation and subsequent increased activity, as measured by increased ATP-binding cassette transporter (ABC) A1 and ABCG1 mRNA expression. Increased cholesterol export resulted in decreased retinal endothelial cell (REC) cholesterol levels (91). SIRT1 activation, in vivo, prevented diabetes-induced inflammation and vascular and neural degeneration (91). Of interest, *Cyp46a1*^*−/−*^ mice have normal fasting blood glucose levels but a 1.8-fold increase in retinal cholesterol. *Cyp46a1*^*−/−*^ mice exhibit venous beading and tortuosity, microglia/macrophage activation, and increased vascular permeability, features commonly associated with DR (90), suggesting a potential role of CYP46A1 in DR pathogenesis (90).

Collectively, disruption of cholesterol homeostasis in diabetes (88, 92) with nonspecific cholesterol entry due to inner BRB breakdown and decreased cholesterol export and cholesterol metabolism to more soluble oxysterols by RPE and neuroretina could lead to increased retinal cholesterol levels contributing to DR pathogenesis.

### Protective effects of retinal PPARα activation in DR

PPARα is expressed in all retinal layers, and in both T1D and T2D models, intraretinal expression of PPARα, but not PPARβ/δ or PPARγ, was significantly downregulated (93). Activation of PPARα by fenofibrate in animal and cell culture models have shown protective antiinflammatory and antiapoptotic effects in the endothelial cells, pericytes, and RPE cells that appear to be independent of lipid-lowering effects (94, 95).

Diabetic PPARα KO mice developed more severe DR, whereas overexpression of PPARα in the retina of diabetic rats significantly alleviated diabetes-induced retinal vascular leakage and retinal inflammation (93), was neuroprotective (96), and prevented pericyte dropout (97). Cell culture studies further demonstrated that PPARα overexpression inhibited endothelial cell migration and proliferation. PPARα overexpression and activation significantly reduced oxidative stress-induced apoptosis, decreased reactive oxygen species production, and downregulated NAD(P)H oxidase 4 expression through blockade of NF-κB activation in primary human retinal capillary pericytes (97). Moreover, in diabetes, PPARα downregulation was associated with endothelial progenitor cell deficiency and inadequate retinal vascular repair (94, 95).

### Ceramide metabolism in DR

Fenofibrate has established lipid-lowering effects and known nonlipid PPARα effects; in addition, lipidomic studies have revealed that fenofibrate treatment leads to a global decrease in ceramide levels (98). Moreover, circulating ceramide levels were shown to correlate strongly with future adverse cardiovascular events such as myocardial infarction and stroke, and ceramides containing the C16, C18, and C24:1 acyl chains displayed a superior independent predictive value for plaque instability and/or future fatality than conventional lipid profile measures, including LDL cholesterol (98, 99, 100, 101, 102). The overall role of sphingolipid metabolism in the retina is discussed in detail by Simon *et al*. in this issue (103).

**Fig. 4:**
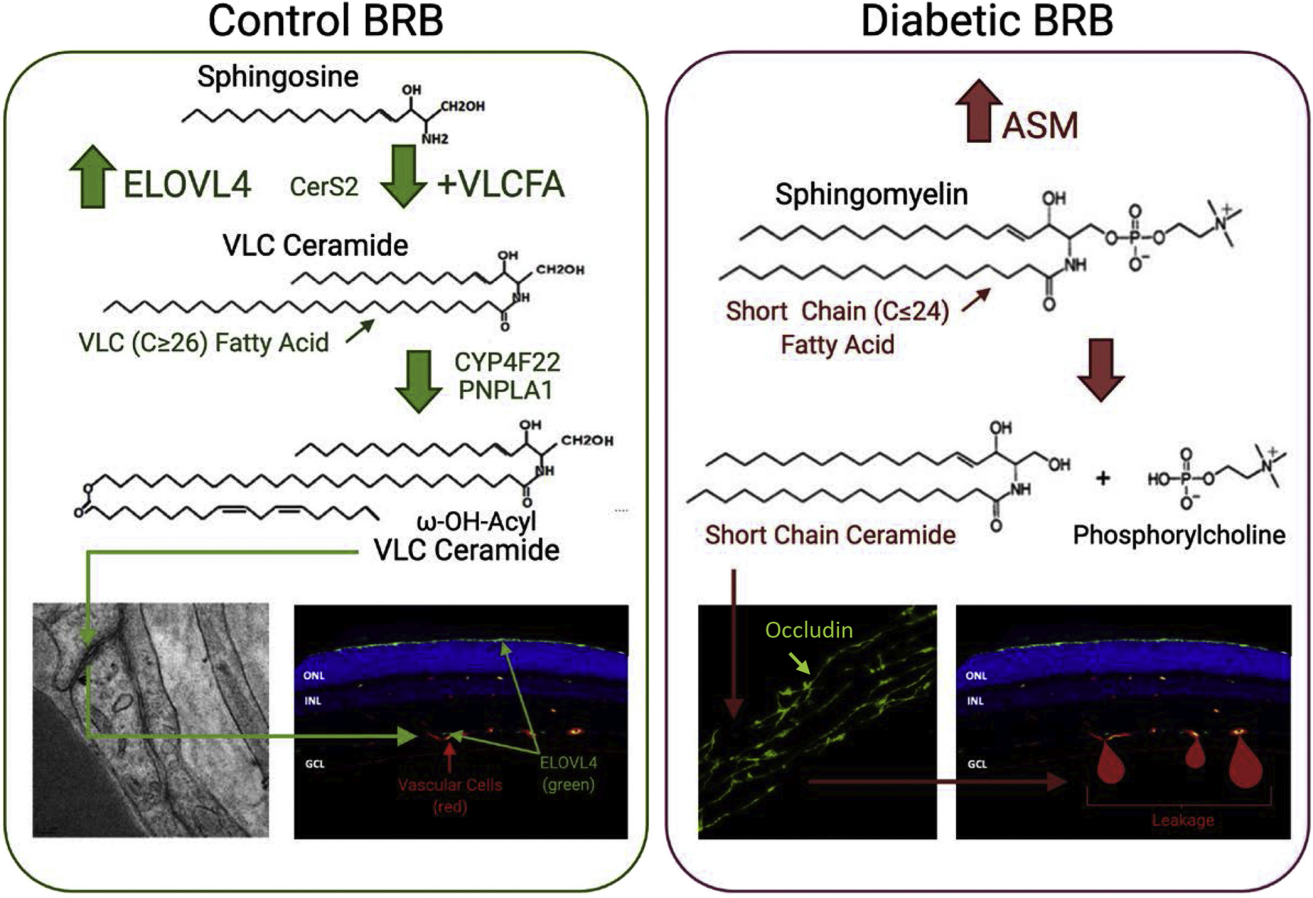
Ceramide metabolism in the retina. In control retina, there is a balance between proapoptotic short chain and probarrier VLC ceramides. In diabetic retina, owing to the reduction in Elovl4 and increase in ASM, the balance is shifted toward proinflammatory ceramides, leading to the breakdown of the tight junction barrier and leaky blood vessels.

Dysregulation of ceramide metabolism in the diabetic retina is well documented in animal and cell culture studies (50, 51, 52, 104, 105). An important example of such dysregulation is the shift in the spectrum of sphingolipid actions from protective, probarrier VLC ceramides (C ≥ 26) to proinflammatory and proapoptotic short-chain (SC) ceramides (C ≤ 24) (Fig. 4). SC ceramides in the retina are mainly produced from sphingomyelins by acid sphingomyelinase (ASM) (50, 51, 52, 106, 107). Of interest, the ceramide species most highly associated with plaque instability, the C16, C18, and C24:1 acyl chain ceramides, are the main products of ASM, and these are the ceramides that are upregulated in the diabetic retina. As fenofibrate has been shown to have a potent effect on the global decrease in ceramides, it would be interesting to explore the effect of fenofibrate on retinal ceramide levels in the presence of diabetes.

Production of VLC ceramides involves elongation of VLC fatty acids protein 4 (ELOVL4)-mediated synthesis of VLC saturated fatty acids that are then incorporated into ceramides by the action of ceramide synthases (CerS) (108). There are six CerS identified as CerS1–CerS6. Each CerS exhibits fatty acyl chain length specificity, as well as tissue specificity. CerS1, 4, 5, and 6 are specific for SC fatty acids, and CerS2 and 3 are specific for long and VLC fatty acids (108). CerS 2 and 3 are expressed in the retina; however, their role in retinal health, as well as in the pathogenesis of diabetic retinopathy, is not well understood.

ASM is highly upregulated (51) and ELOVL4 is downregulated (53) in the diabetic retina. Downregulation of ASM or upregulation of ELOVL4 were protective against diabetes-induced retinal vascular degeneration in cell culture and animal models (51, 109).

Dysfunctional sphingolipid metabolism in the diabetic retina, with an increase in SC ceramides due to pathological activation of ASM and a decrease in VLC ceramides due to downregulation of ELOVL4-mediated production of VLC saturated fatty acids, is an important metabolic insult contributing to the development of DR (50, 51, 52, 106, 107). The role of ASM-mediated proinflammatory and apoptotic signaling is well studied (50, 51, 52, 89, 104, 105, 106, 110, 111, 112), but the possibility of a protective and probarrier role of ELOVL4-mediated upregulation of VLC ceramides in the retina remains largely unexplored.

ELOVL4 is the most highly expressed elongase in the retina (53). ELOVL4 dysfunction is associated with Stargardt-like macular dystrophy (STGD3) and the enzyme was recently found to be downregulated in the DR model (53, 109, 113). Fatty acids with ≥C24 chain are used as precursors by ELOVL4 for synthesis of ≥C26 fatty acids. Although the highest retinal expression of ELOVL4 is found in the photoreceptor inner segment (114, 115), it is also expressed in other retinal layers (116, 117), including REC and RPE cells (109). The product of ELOVL4-mediated elongation will depend on the precursor fatty acids. As the photoreceptors are rich in omega-3 PUFAs, the main products of ELOVL4 in photoreceptor cells are C32–36 PUFAs. Endothelial and epithelial cells have a much lower n3 PUFA and higher saturated fatty acid content leading to higher VLC saturated fatty acid production that in turn provides substrates for CerS 2 and 3 (118, 119).

Intact BRBs are essential for normal retinal function, and BRB breakdown represents an important initiating factor in the pathogenesis of DR. Both the inner REC and outer RPE cell layers contribute to the formation of the BRB (120). Tight junctions (TJs) are important components of this barrier and loss of TJ integrity leads to increased permeability and barrier breakdown. A key role for lipids in maintaining TJ and barrier integrity has been postulated for a long time (121, 122, 123, 124); however, our understanding of the role of lipids in barrier function is still limited. We have recently demonstrated that ω-OH acyl-VLC ceramides are present in the TJ of BRB (109). Moreover, ELOVL4 overexpression and VLC ceramide production prevented diabetes-induced BRB breakdown and normalized the TJ structure and function in the diabetic mouse model and inhibited vascular endothelial growth factor-induced permeability in a bovine REC model (109).

Recent studies demonstrate, in addition to cell membrane and TJ effect of ceramides, that there is an intricate connection between ceramide and mitochondrial function. Mitochondria play a cornerstone role in cellular metabolism, and even a slight modification of mitochondrial function can lead to pathology. Indeed, mitochondrial damage precedes histopathological abnormalities in DR in T1D and T2D models (125, 126, 127) with mitochondrial fragmentation (128, 129, 130), swelling and loss of cristae, epigenetic changes in mitochondrial DNA, reduction of transport proteins (126, 131, 132, 133), and mitochondria-ER regulation (128) in REC in diabetes. In addition, impairment of RPE mitochondria is associated with increased oxidative stress, reduced ATP, and compromised autophagic and phagocytic capacities (134). Impairment of neuronal mitochondria contributes to the loss of retinal synapses and neuronal cell death in diabetes (135, 136).

Mitochondria have been shown to contain many sphingolipids, including sphingomyelin and ceramide (137, 138), as well as enzymes of the sphingolipid pathway, including ceramide synthases (CerS1, CerS2, CerS4, and CerS6) (139, 140, 141, 142, 143), acid (144) and neutral sphingomyelinases (145, 146), and neutral ceramidases (140). Ceramide-induced restriction of respiratory chain function at the level of Complex III, as well as succinate accumulation, has been identified as a causative factor in ischemia/reperfusion and stroke-induced tissue damage (147, 148).

Ceramides were shown to have effects on respiratory enzymes and in addition contribute to mitochondrial outer membrane permeability either through S1P and hexadecenal production and activation of BAX/BAK or directly through the formation of protein-permeable ceramide channels in mitochondrial outer membranes (149). These channels are shown to play a key role in the induction of apoptosis through the release of cytochrome *c* into the cytoplasm (150).

A recent study demonstrated that mitochondria isolated from streptozotocin-induced diabetic rat retinas have an increase in ASM-mediated SC ceramide species production and in the ceramide-to-sphingomyelin ratio compared with controls. Moreover, RPE cells derived from diabetic donors showed fragmented mitochondria and decreased respiratory control ratio (151). The respiratory control ratio was corrected by ASM inhibition, showing that a diabetes-induced increase in mitochondrial ceramide through an ASM-dependent pathway contributes to impaired mitochondrial function and retinal pathology in DR (151).

Overall, dysregulation of sphingolipid metabolism in the diabetic retina appears to be an important and largely unexplored aspect of DR pathogenesis, one that could aid the discovery of novel therapeutic targets for DR prevention and treatment.

## Concluding Remarks

Clinical and basic studies of the last decade removed any doubt that lipid metabolic abnormalities play an important role in the pathogenesis of DR; however, lipid-based treatment strategies are just beginning to emerge. Fibrates and omega-3 PUFAs have a long history of showing protective effects against diabetic retinopathy; however, the clinical data on fenofibrate, although quite strong, come from cardiovascular trials with incomplete retinal analysis. The Fenofibrate And Microvascular Events in Type 1 Diabetes Eye (FAME 1 EYE) study is an ongoing fenofibrate clinical trial with DR as the primary outcome. The results of this study will further improve our understanding of the mechanism(s) of retinal effects of fibrates. Less information is available on the effects of statins: possible protective effects have been suggested in retrospective studies in Denmark, Taiwan, the United States, and Japan; however, without the data from large randomized, placebo-controlled DR clinical trials, the benefits of statin use remain uncertain.

The complexity of lipidome and tissue and organelle-specific lipid effects add to the challenge but at the same time provide a plethora of promising unexplored approaches to harness new knowledge of intraretinal lipids and lipoproteins to find a cure for this most feared diabetic complication.

## Conflict of interest

The author declares that they have no conflicts of interest with the contents of this article.
